# An Internet Based Intervention for Adults With Autism Spectrum Disorder—A Qualitative Study of Participants Experiences

**DOI:** 10.3389/fpsyt.2021.789663

**Published:** 2021-12-22

**Authors:** Britta Westerberg, Sofie Bäärnhielm, Clover Giles, Ulrika Hylén, Fredrik Holländare, Susanne Bejerot

**Affiliations:** ^1^University Health Care Research Center, Faculty of Medicine and Health, Örebro University, Örebro, Sweden; ^2^Center for Psychiatry Research, Department of Clinical Neuroscience, Karolinska Institutet, Stockholm Health Care Services, Stockholm, Sweden; ^3^Center for Lifespan Developmental Research, Örebro University, Örebro, Sweden; ^4^Department of Psychiatry, School of Medical Sciences, Örebro University, Örebro, Sweden

**Keywords:** patient experience, content analysis, cognitive behavioral treatment, interview, autism spectrum disorder

## Abstract

**Background:** Adults with autism spectrum disorder face several barriers to accessing evidence-based care, including difficulties in communicating needs, social anxiety or in traveling to a health care unit. In recent years, several forms of internet-based treatments have shown to be effective for a variety of psychiatric conditions. Internet-based treatment alternatives allow convenient and flexible formats, and therefore have the potential to increase access to health care for individuals with autism spectrum disorder. However, knowledge about how internet-based treatment features may suit the needs of individuals with autism is limited. The aim of this study was to explore the participant experiences of an internet-based intervention for adults with autism spectrum disorder. The primary focus of the investigation was on autism-specific needs in relation to the features unique to the online format.

**Methods:** In this qualitative study, semi-structured telephone interviews were conducted with 14 participants who had completed a text-based internet-based intervention for adults with autism spectrum disorder. We used an inductive approach and analyzed the data using qualitative content analysis.

**Results:** Five main categories were identified: (1) implications of the online format, (2) the fixed non-individualized model, (3) therapist interaction, (4) interacting with other participants, and (5) making use of the treatment content. Overall, participants appreciated the availability and that they could work on their treatment independent of time or location. Among those participating in group-based chat-sessions with the other participants, it was considered a generally positive experience. Furthermore, most participants felt safe and relaxed in relation to the therapist and appreciated the text-based format. However, several participants felt that the format and content of the treatment was not sufficiently adapted to their individual life situation.

**Conclusion:** In conclusion, this internet-based treatment constitutes an accessible and energy-saving treatment alternative for adults with autism. Further, integrating group-based components seems feasible in an otherwise individual internet-based treatment for individuals with autism. However, group-based components do require a clear purpose and rationale. Future studies should develop and evaluate treatment adaptations tailored to individual needs.

## Introduction

Autism spectrum disorder (ASD) is a neurodevelopmental disorder characterized by disabilities in two major areas: (1) social interaction and communication and (2) restricted and repetitive patterns of interest, behavior and activities (1). Numerous studies have evidenced that psychiatric comorbidities with anxiety disorders and depression are common (2–8), and one meta-analysis concluded the prevalence of any psychiatric comorbidity to be 54.8% (4). Life quality is generally reported to be low among adults with ASD (9, 10) and suicidality have shown to be over-represented among high functional individuals with ASD (11).

The number of patients diagnosed with ASD is increasing (12, 13), but health care services are often unable to meet their needs (14, 15). Autism-specific factors, such as difficulties communicating needs or maintaining structure (14, 16) are related to the inaccessibility of health care. The advantages of group-based interventions for individuals with ASD have been suggested in several studies (17–20) but there are several barriers to participation. Some people with ASD refrain from using public transport to health care facilities due to perceptual sensitivity (15). Others avoid contact with health care services as it involves communicating with, and describing their needs to a stranger, which may be perceived as a stressful experience. Other barriers to health care are related to the provider or system deficiencies, such as lack of knowledge about autism, inaccurate beliefs about patient needs, the use of inaccessible language or limited number of available health care alternatives (21).

Moreover, negative experiences with health care services may have serious consequences for the individual's mental well-being. According to Cassidy (22), repeated experiences of unmet needs contribute to the high suicidality rates among autistic adults. Considering the obstacles to utilizing health care, treatment alternatives should therefore be adapted to meet the specific needs of the group (23, 24).

In recent years, a considerable body of research has emphasized the advantages of technology-based solutions to facilitate communication and learning for individuals with autism (25). A recent review investigating the use of technology and web-based services in educational settings showed that technological solutions may be beneficial to people with ASD as they provide a comfortable environment that promotes learning (26). Furthermore, people with autism have been seen to prefer computer-mediated communication more than others, partly because it allows for more thinking time (27) and increases control over their communication (28). The slower pace in online communication is suggested as one of the most important reasons for this preference, which may explained by decreased demands on the ability to process information (27). Further, written computer-mediated communication obviates the social stress surrounding conventions related to e.g., body language, eye contact and tone of voice, all of which could be very distressing for a person with ASD.

An increasing number of studies support the efficiency of internet based treatment (IBT) targeting a variety of psychiatric conditions such as depression (29), panic disorder (30), generalized anxiety disorder (31), social anxiety (32), Attention Deficit Hyperactivity Disorder (ADHD) (33) and psychosis (34). IBT has also shown to be useful in treating psychiatric comorbidities among children with ASD (35, 36). Even compared to face-to-face treatment, IBT has shown to be effective (37).

IBT alternatives are available in different forms, depending on target group, therapeutic method and specific health issue, but common for all IBTs is that the location is optional for the patient. Commonly, IBTs are exclusively individual, i.e., no contact with other patients, and the research on group-based components in internet-based interventions is scarce.

One area of interest in IBT research is the presence of a therapist/coach vs. an entirely self-guided approach, where no coach or therapist is involved. Several studies have highlighted the importance of therapist guidance and coaching in internet-based interventions (29, 38–41). The communication between patient and therapist is written, and can be both immediate (synchronous)—in a chat-like manner—and independent of timing (asynchronous)—similar to e-mail (42).

IBT can also be tailored, meaning that patients can be prescribed different therapeutic components based on their individual needs. However, more common is a disorder-specific approach where the therapeutic components provided are the same for all patients following the same treatment manual. Research comparing these two approaches suggests that they have similar effects (43, 44).

In studies evaluating the user experience of IBT, participants commonly consider it to feel safe and enables a sense of control over one's own treatment (45). Further, most participants appreciate the flexibility that the online format entails regarding time and location (45–48). However, although experiences of IBT for psychiatric conditions are generally positively evaluated, some hindrances are repeatedly described. Commonly noted is a lack of individualization of the treatment content (40, 41, 45, 49) even for interventions denoted as individually tailored (50). Others report insufficient therapeutic support (45) or describe a feeling of being left alone in their treatment (46). Ex-participants' own suggestions for improvement of IBT often involve increased individualization and flexibility in delivery (e.g., combining IBT and face-to-face treatment), and improved technology (40, 45).

As difficulties in direct social communication is depicted as a barrier to seeking and accessing health care, the indirect and time allowing structure of online communication—as offered in internet-based care—would be highly suitable for individuals with ASD. Still, the research on IBT for individuals with ASD is limited. In one exception, Backman (51) explored the feasibility of an internet-based psychoeducative program for young adults and adolescents with ASD. Clinicians reported good feasibility and participants reported general satisfaction with the program. In another study, Sehlin (52) investigated the experiences of young adults and adolescents with ADHD and/or ASD who had participated in an internet-based support and coaching program. Participants emphasized the important role of the coach/supervisor, as well as the convenience of communicating through written word and staying at home.

We are not aware of any previous studies investigating experiences of participating in IBT among adults with ASD. As such, there is little known about the fit between autism-specific difficulties and the features unique to internet-based interventions. Nonetheless, knowledge about the needs and preferences of treatment components among individuals with ASD is crucial for the development of feasible and effective programs.

For the reasons mentioned above, the aim of this study was to explore the experiences of adults with ASD that have participated in an individual IBT including some group-based components. The primary focus of the study is on autism-specific needs in relation to the features unique to the IBT format.

## Methods

### Design

This qualitative interview study is part of a project evaluating an internet-based cognitive behavioral treatment (ICBT) for people with ASD, carried out during the spring of 2019. In this study, semi-structured interviews were conducted to capture participants' in-depth thoughts and reflections. We used an inductive approach and analyzed the data using qualitative content analysis (53).

### Intervention

The *Method for Internet based treatment for Life quality for adults with Autism Spectrum disorder* (MILAS) is an 18-week individual ICBT for adults with ASD. The aim of the treatment is to improve quality of life through enhancing function, self-acceptance and self-knowledge. MILAS is based on a comprehensive group-based CBT manual, ALMA, for adults with ASD (19, 54). During October 2017–December 2018, the manual was converted into an internet-based intervention—named MILAS. Group exercises were individualized, information otherwise given verbally was written down, instructions were rephrased to address the patient directly instead of the group leader and the exercises were adapted to a home environment.

The patients undergoing MILAS are assigned weekly text-based modules (sections) covering the themes presented in Table 1. All texts (in total 41,000 words) are also audio-recorded and available for listening. The patients complete individual exercises according to the current module, after which a therapist provides feedback on the completion of each exercise. The patient-therapist communication is asynchronous, and the patient is free to write to the therapist at any time. After 7 days of inactivity in the treatment, the patient receives a message reminding them of their treatment. In case of continued inactivity, the therapist provides additional reminders.

**Table 1:** Weekly themes of the internet-based CBT MILAS.

1	Introduction and diagnostic criteria	10	Depression
2	Individual goals	11	Social anxiety
3	Behavioral analysis	12	Central coherence
4	Mentalization	13	Relations
5	Social interaction I	14	Stress & sleep
6	Social interaction II	15	Organization at home
7	Problem solving	16	Employment
8	Emotions & body language	17	Diet & physical exercise
9	Perception	18	Summary and evaluation

The treatment is disorder-specific, focusing on common and conventional strategies for managing difficulties among individuals with ASD. However, there are a few elements of individual tailoring. Most apparent, in module 2, where the patient selects an individual goal, which is intended to influence how the patient adopts and makes use of the forthcoming exercises.

MILAS combines individual exercises with group-based components. Every second week, the patients have the opportunity to participate in recurrent group-based chat sessions (eight in total) with other patients. These are moderated and led by the therapist, and focus on the themes of the two most recent modules.

Within the project we conducted a RCT evaluating the feasibility and effectiveness of MILAS. Forty-two adults diagnosed with ASD participated in the treatment group. The participants were divided into chat groups each consisting of seven participants. However, only 2–5 people usually participated in each session. Four therapists/supervisors were involved in the RCT, whereof three were clinical psychologists and one was a registered nurse.

### Participants and Recruitment

**Figure 1:**
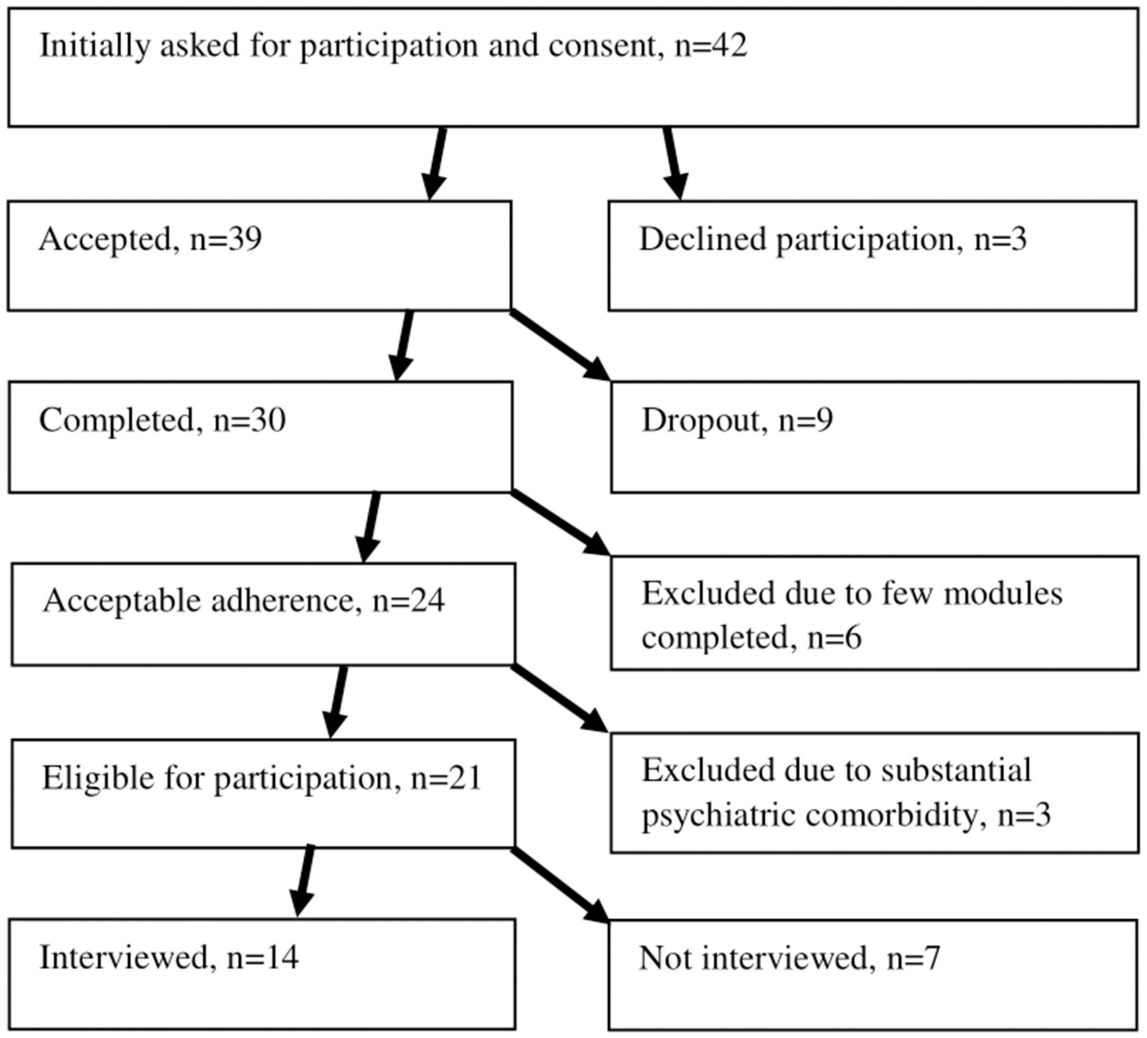
Flowchart.

The participants of this qualitative interview study were 14 adults with ASD who had participated in the RCT of IBT MILAS. Before commencement of the IBT, all participants (*N* = 42) were asked if they were willing to be interviewed after the treatment had ended. Thirty-nine participants consented to participation. Reasons for declining participation were not shared. We used a purposive sampling method to ensure involvement of information-rich cases. Thus, to be eligible for the interview study, the two following inclusion criteria had to be met: (1) having completed sufficient number of modules considered necessary to attain a thorough experience of the intervention (eight or more) and (2) that the individuals' autistic characteristics were not overshadowed by other psychiatric disorders such as a pervasive personality disorder or current severe symptoms of depression. See Figure 1 for recruitment process.

Of the 21 eligible participants, we alternated our reaching out-attempts (phone calls) between participants from different chat groups and with different rates of attendance in the chat sessions, to ensure a breadth of experiences and to model the RCT sample. After 14 interviews, no new responses were forthcoming and therefore, the information obtained was judged as sufficient to answer the research question. Thus, a decision was made not to conduct more interviews.

Five participants were male and nine were female. Age ranged between 21 and 55 years. Four of the interviewed participants did not participate in any of the chat occasions. The reasons for not participating in the chat sessions—or only passively participating (observing) were (1) feelings of discomfort associated with interacting with several strangers at a time or (2) the chat sessions were scheduled during working hours. All participants filled out the RAADS-14 Screen, a validated 14 item self-rated instrument that measure autistic traits. Previous research has shown a median score of 32 in participants with ASD (55). Clinical and demographic characteristics of the participants are shown in Tables 2, 3.

**Table 2:** Characteristics of participants.

**ID**	**Gender**	**Age**	**Occupation**	**Habitation**	**Modules^b^**	**Group chat^c^**
3	Male	20–24	Sick leave	Parents	17	No
9	Female	20–24	Employed	Parents	18	No
10	Female	20–24	Other	Partner	16	A few times
11	Female	20–24	Student	Parents	11	Yes
1	Male	25–30	Other	Other	17	Yes
4^a^	Male	25–30	Unemployed	Parents	15	Yes
13	Female	25–30	Daily activity	Alone	18	No
12	Female	30–34	Other	Alone	17	Yes
7	Female	35–40	Employed	Partner/children	18	A few times
8	Female	35–40	Unemployed	Parents	18	Yes
14	Male	35–40	Unemployed	Alone	18	No
2	Female	40–44	Other	Partner/children	16	Yes
6	Female	40–44	Unemployed	Partner/children	18	A few times
5^a^	Male	55–60	Unemployed	Partner/children	9	Yes

a *Interviewed face to face*.

b *Number of treatment modules completed (max 18)*.

c *Taking part in the 1 h group-based chat-sessions held every other week (max 8)*.

**Table 3:** Means and proportions on significant demographic and clinical variables.

Age, M (SD)	32.5 (10.2)
Sex, % female	64.3
Psychiatric comorbidity, %	42.9
Employed, %	14.3
College/university degree, %	21.4
Experiences of bullying, %	78.6
RAADS-14, median	31
N of modules (SD)	17.6 (0.6)

### Data Collection

The interviews were conducted in Swedish, by telephone (*n* = 12) or face-to-face at a local habilitation clinic (*n* = 2), ranging between 33 and 89 mins (mean = 54) and were performed by BW (12 interviews), SB (two interviews) and CG (two interviews). The three female interviewers were; a PhD-student and licensed clinical psychologist, a senior psychiatrist and psychotherapist, and a Master of Science in Clinical Psychology. BW and CG had previously been therapists in the intervention, thus did not perform interviews with participants they had treated themselves. Prior to the interview, participants were sent a list of the treatment modules, and suggested to have the list in front of them to support their recollection during the interview.

The interview was semi structured and followed an interview guide with open-ended questions. It covered the following areas; (a) the participants' experiences of the particular intervention, (b) the impact the intervention had on their perception of one's self, others or their ASD-diagnosis, (c) their experiences of different aspects related to interacting with their therapist or other participants online, and (d) other thoughts and preferences they had regarding the design of the treatment. Exploratory questions were asked if needed.

It is unknown whether participants were alone during the telephone interviews. During the on-site interviews, only the interviewer and interviewee were present. All interviews were conducted between 1 week and 2 months after the end of the intervention and no repeated interviews were carried out.

The interviews were audio-recorded and transcribed verbatim, and all data analyzed is based on the transcriptions. Four interviews were transcribed by the lead author (BW), and the remainder were transcribed by a transcription company.

### Data Analysis

The analysis was made using conventional content analysis (53) in order to capture the variety of personal experiences. QSR Internationals Nvivo 12 software was used for coding and categorizing of data. The international standard checklist for reporting qualitative research, COREQ (56), was followed in order to report the research process in a transparent and comprehensive way.

Primarily, all transcripts were read through in order to become familiar with the data. Meaning units were then highlighted and given a describing code in Swedish. Meaning units with the same meaning were grouped into one code. The codes were then sorted into sub-categories. The fit between codes and categories were regularly scrutinized by going back and forth between the stages. Out of the sub-categories, a few main categories could be identified. Coding was done using an inductive approach, i.e., categories were derived exclusively from the data. BW held the primary responsibility for coding of data. Categories and sub-categories were then examined by, and discussed with SB. The categories and sub-categories were translated into English during the reporting phase. To ensure that participants' true perspectives are represented and not omitted or misinterpreted, the results of the analysis, and an inquiry for feedback, were sent to all participants prior to submission. No feedback from participants was received.

## Results

### Findings

Altogether, 274 codes and five distinct categories were identified within the data, including 2–3 subcategories each. The five categories are (1) implications of the online format, (2) the fixed non-individualized model, (3) therapist interaction, (4) interacting with other participants and (5) making use of the treatment content. See Table 4 for a detailed description including subcategories and examples of codes.

**Table 4:** Categories, subcategories and codes.

**Categories (Number of occurrences)**	**Subcategories (Number of occurrences)**	**Codes (examples)**
Implications of the online format (158)	Convenient and accessible (95)	Optional time
		Saves energy
		On my own terms
	Communicating through the written word (30)	Enables more time to think
		Easier expressing oneself
		Better reading than listening
	Limitations to the online format (33)	Wanted to see the therapist
		Requires more discipline
		Stressed by technical problems
The fixed non-individualized model (163)	No individual customization of format (69)	One week/module is too short
		The chat was too infrequent
		Wanting more communication with therapist
	Preferring optional content (50)	Some modules were pointless
		Too broad variety of modules
		Couldn't relate individual goal to modules
	Appreciating the fixed format (44)	Appreciating getting a reminder
		Became a routine
		18 weeks was appropriate
Therapist interaction (63)	Experiences with the therapist contact (41)	The feedback was rewarding
		Felt safe
		Like having someone watching over you
	Preferences of therapist qualities. (22)	Understanding
		Well-developed language
Interacting with other participants (63)	Challenges with interacting with others (18)	Felt uncomfortable To many new individuals
	The meaningfulness of interacting with other participants (45)	Got to know other perspectives
		Got tips from others
		Discussing with others with similar problems
Making use of the treatment content (164)	Personal change (83)	Gained higher self confidence
		Have stopped over-analyzing
		Gained increased well being
	Specific modules and strategies (51)	Mentalization module useful
		Social interaction module useful
		Depression module useful
	General positive evaluations (30)	Easy understandable texts
		Useful for the future

#### Implications of the Online Format

This category covers aspects that are related and unique to the online format. All participants viewed aspects of the online format as more or less convenient, and well fitted to their preferences. However, a majority also noted potential negative aspects of the fact that the treatment was exclusively carried out over the internet. All participants reflected on how written communication affected the therapist relationship, the ability to assimilate the material and express oneself.

##### Convenient and Accessible

Almost all participants emphasized the advantages of freely choosing when to work with the material, and the convenience of being able to stay home, where one feels safe and comfortable.

“*I need to, like, be in an environment that feels safe and calm and that I'm… that it's at a time of day when I … have energy and … like, the strength to do it. And I could control all of that myself, and I really liked that. It gave me a greater ability to make the most out of this treatment.”* [Participant 6]

They described several benefits of not having to plan their visit to the health care facility—e.g., not having to look up bus schedules, estimate time expenditure or take time off from work. The avoidance of uncomfortable traveling saved energy that instead could be dedicated to the therapy. Not having to meet face to face and focus on body language, tone of voice or eye contact was also experienced as saving energy.

“*Just because you see the person, I don't think it makes it more personal, it may even be [that I put] more focus on my body language and making eye contact than on what the person is actually saying, I think.”* [Participant 3]

Comparisons with regular health care were frequently made. Participants emphasized that engaging in the internet-based intervention was substantially different from their usual experiences of accessing health care. While regular health care was described as a troublesome process, they experienced the internet-based format to be “on their own terms.”

“*In my experience, if you have autism it's like: planning of time, making it there, getting to a meeting, describing your problems, charting them and getting help—it's a process that is… you give up. I've been through it so many times and I find it so hard. You continue living with your problems instead of seeking help because getting help is such a long process.”* [Participant 7]

Five participants described how the online format allowed the opportunity to involve a relative or caretaker in their treatment process. Some invited a family member to read parts of the material in order to discuss the information, aiming to increase their own and their relatives understanding of their disability.

“*I mean I could log in and read as many times as I wanted to. And if it was something I felt ‘Ok, I really don't understand this’, then I could give my phone to my mother and ask her how she would have interpreted it.”* [Participant 13]

*Communication through the written word* is related to the feeling of convenience described above. Most participants appreciated the possibility to think through and revise their texts before submitting them. This gave many a sense of control over what was said, as opposed to verbal communication which was described as more direct and giving little room for corrections. The ability to think through their statements was described as stress reducing. Participants also appreciated that the feedback from therapists was written, as it was possible to re-read it several times, and thereby deepen their understanding of it.

“*Because in written word you can thoroughly read through what she [the therapist] wrote to you and can take much more time to come up with a good answer, and then you may change you answer several times before you send it if you're not satisfied with how you phrased it. But when you talk, you said what you said and then you have to stand for it. (…) In that sense, I felt no pressure, because I knew I had time to formulate what I really wanted to say. I appreciated that.”* [Participant 9]

*Limitations to the online format* were described by some participants. This sub-category covers both aspects related to the technological components and aspects described in contrast to a face-to-face-meeting. A recurring experience was that technological problems resulted in feelings of stress and irritation. Moreover, some thought that the internet-based platform had suboptimal configuration.

One participant expressed a feeling that the therapist was more distant compared to in real life encounters. A few participants would also have preferred some verbal contact with the therapist, e.g., some face-to-face meetings or a picture of the therapist.

“*One disadvantage is that the therapist doesn't see the patient. Because when you see someone, you can get clues about how they feel. And then if someone feels bad in this kind of treatment, there is a risk that it remains unnoticed if the person doesn't express it very clearly.”* [Participant 6]

One participant thought that the risk of being distracted by other issues was higher at home compared to an onsite session, and others experienced difficulties initiating the exercises. Many participants experienced the online format as heavily depending on their own responsibility. This was described as a difficult undertaking—often attributed to ASD-related issues with planning and engaging in new tasks.

“*I think it's better being there [at a health care facility] because then you can't distract yourself in the same way. I can't just start my computer games because there are no games there. But if I sit in front of my computer, it's just one click away, starting a game or watching YouTube and just disappearing. (…) The advantages are that you can work the way you feel comfortable, without demands. And the disadvantages are that you may get TOO comfortable, you distract yourself with things and end up doing nothing.”* [Participant 4]

#### The Fixed Non-individualized Model

This category covers experiences regarding the fixed format and limitations for individual adaptations. For example, that new treatment content was presented weekly regardless of whether previous modules were completed, and that all participants were provided with identical modules, regardless of level of disability, psychiatric symptoms or demographics such as age or social situation. Furthermore, suggestions about changes in format or content were presented by several participants.

*Preferring optional content* refers to the inability and desire to deselect, choose or adjust the content of treatment modules. A majority of the participants experienced that some components were irrelevant to their specific life situation. Some participants reasoned that this might be due to the practitioners' desire to create an intervention that suited everyone. Participants described some modules as “pointless” or “fruitless”, due to inadequate level of complexity of the material, that they already were familiar with the topics, or that the exercises were unnecessary.

“*It would have been good if you could deselect some themes, adjust to the individual, and their own situation. For those with children, it would have been interesting with exercises more related to having children, for example. (…) So that you don't get a treatment that is too broad, with too many questions and suggestions, so that you can relate [to the treatment] a little better.”* [Participant 7]

##### No Individual Customization of Format

Twelve of 14 participants expressed more or less negative views on the inability to make individual customizations of the format. The most recurring comment was that the chat session with other participants was scheduled too infrequently (every second week) or at the wrong time of the day (4 pm). Some participants found that the regularity of new treatment material gave them a feeling of stress. Rather, some participants would have preferred that modules were provided according to their individual (irregular) pace. Furthermore, some participants were not satisfied with the limited amount of contact with the therapist and desired more frequent contact.

##### Appreciating the Fixed Format

Almost all participants recalled more or less advantageous aspects of the fixed treatment format. This was often related to the convenience of not having to take responsibility over the format, that it felt safe and became a natural element of the week. They appreciated the reliability and predictability that new content was presented on a routine basis. Furthermore, the length of the treatment-−18 weeks—was generally considered sufficient.

“*This whole MILAS-project became a part of me, like, a part of my life. It went on for so long, it sort of became natural. As natural as brushing your teeth, if you know what I mean?”* [Participant 8]

#### Therapist Interaction

This category covers both the participants' experiences of interacting with their therapist online and their views regarding the skills and characteristics desirable in a therapist working with internet-based interventions. Most participants reviewed the presence of an individual therapist as an important part of their treatment and emphasized the importance of the therapist expressing understanding and concern for the participant.

##### Experiences of the Therapist Contact

Several participants experienced the feedback as rewarding and confirming, and felt safe in the therapeutic relationship. Others appreciated that the feedback provided useful tips, or a wider range of perspectives. Some participants also reflected over the individual customization of the feedback in contrast to the general content of the modules.

“*Because the modules were adapted to fit everyone and the feedback was adapted to me, it was individualized, that's a huge difference.”* [Participant 2]

However, the messaging function was used in a variety of ways by the participants. Some used it to enable a deeper conversation with the therapist while others felt uncertain as to whether it was okay to initiate a conversation with the therapist or even respond to the feedback.

“*It was useful to get reassurance that what you're thinking about isn't so weird. Or ‘you’re on the right track, continue’. I‘ll miss that a bit. It was nice to have a contact online, to be able to write to the therapist and get feedback. To have somebody watching over you.”* [Participant 7]

##### Preferences of Therapist Qualities

A majority of the participants clearly stated that a deep willingness to understand participants is essential when working online. Other traits perceived as important in a therapist were flexibility, expertise within the field, well developed mentalization skills, a direct communication style, honesty, linguistic competence, and an ability to think outside the box.

#### Interacting With Other Participants

This category refers to statements regarding the experience of interacting with other participants through a biweekly scheduled chat session. As only 10 of the 14 participants had any experience of the biweekly chat sessions, the lived experience emanated from these. The other participants shared their subjective reasons for not participating in the chat.

##### Challenges With Interacting With Others

Some participants expressed fears regarding interacting with others about potentially personal subjects. Others experienced interacting with other people as generally difficult, stressful or socially uncomfortable. Some would have preferred fewer participants in the chat, or a more structured framework. For some, these factors were explained as reasons not to take part in the chat.

##### The Meaningfulness of Interacting With Other Participants

Participants emphasized the benefits of being exposed to other perspectives, positive aspects of talking to others with similar problems, and sharing useful tips through the chats. Two participants expressed that the forthcoming chat sessions gave them motivation to go through the modules and do the exercises. Some participants would have preferred chats with more participants since it implies more “flow” in the discussion. Others viewed the chat as a good opportunity to practice communication skills.

“*It was very rewarding because I recognized a lot of myself in the others, how they felt and thought. (…) I think it worked fine, if another person wrote something I could write ‘Yeah, that’s exactly how I feel too' and elaborate a little. When I wrote something, it felt like many of the others replied that they felt or thought the same way.”* [Participant 12]

#### Making use of the Treatment Content

This category refers to how participants felt they benefited from the treatment. It involves both self-perceived personal change (e.g., improvement of specific skills, increased well-being, new insights and increased knowledge), and evaluations regarding specific and general components of the intervention that participants perceived to be helpful or rewarding.

##### Personal Change

Thirteen participants reported self-perceived personal change or increased functioning due to the treatment. Some participants felt they were able to handle specific psychiatric symptoms better—such as increased ability to ignore obsessions, improved coping with anxiety and limiting impulses to over-analyse. Some gained strategies to deal with social situations through an increased understanding of others, more frequent participation in social activities and a reduced need to imitate or adapt to other people.

“*Maybe I don't view them [other people] as being as perfect as I thought they were. And—maybe this sounds a bit brutal—but I feel like I've been able to let go of needing to imitate others all the time. I can just think ‘they’re only human and I'm only human, and I don't know them, I don't have to be like them'. Before it was like, neurotypicals were more or less “perfect” and a norm to live up to, but now I just feel like ‘No, I don't have to do that, I can run my own race'.”* [Participant 13]

Some participants reported a general increase in personal well-being due to the treatment, and other reported increased sleep quality, confidence, self-acceptance and positive thinking. The most frequently recurring comments concerned gaining greater understanding and knowledge of themselves and others. However, two participants reported that the treatment did not have any effect on their well-being or on their view of themselves or others.

“*I think mainly that I care less about what people think about me, their opinions of me in general. I'm not super focused on acting as normal as possible anymore.”* [Participant 3]

##### Specific Modules and Strategies

Fifteen out of 18 modules were mentioned as especially useful at least once. The most frequently mentioned were 4—mentalization, 5 and 6—social interaction, 10—depression and 17—diet and physical exercise. The three modules not mentioned were 1—introduction, 16—employment and 18—summary. Five participants found all modules useful in some way.

##### General Positive Evaluations

Several participants expressed that the content was interesting and easy to understand. Many described looking forward to the next module, that they gave tips learnt through the program to others and that what they learnt could be useful in the future.

## Discussion

### Principal Findings

The aim of this study was to explore the experiences of adults with ASD who have participated in an ICBT. The analysis primarily focuses on how aspects unique to the internet-based format correspond to the prerequisites, preferences and needs of individuals with ASD. Using an inductive approach to qualitative content analysis, we found an overall satisfaction with the internet-based format. The most pronounced findings are an appreciation of the accessibility and flexibility of the format, confirming previous research on IBT, both for individuals with ASD (51, 52) as well as other conditions (45–48). Interacting with other participants through the biweekly chat sessions was also considered a generally positive experience.

Several participants evaluated aspects of this treatment as preferable to previous health care experiences. This strengthens the existing picture of negative experiences and insufficiency in health care options/access for adults with ASD. Perceived advantages of online care were described both in relation to individual preferences and to health care providers and services. For example, the online format evoked fewer feelings of stress, which enabled a greater focus on the treatment, and the therapists were perceived to be committed and competent.

The desire for more individualization echoes previous studies on participant experiences of IBT (40, 45, 50), and demonstrates the great variety of needs in the group. The treatment includes many themes, and not all themes were expected to suit everyone. Nevertheless, this breadth is what makes the treatment suitable for targeting everyday challenges of a highly diverse group. Acquiring individualization through tailoring is an obvious option. However, the advantages of group-based components—e.g., the chat sessions of the current intervention—would be hard to acquire in an individually tailored treatment. Further, considering that disorder-specific treatments show similar effects as tailored alternatives (43, 44), the utility of tailoring is not obvious for the objective of this kind of treatment. In addition, the use of only one treatment “path” may allow the therapist to become more familiar with the treatment, thereby increasing their confidence in making appropriate individual adaptations to be delivered in a pedagogical way within the existing framework.

Many of the perceived advantages of the online format relate to energy saving aspects, as it is considered comfortable and require less effort than onsite visits. Several participants appreciated not having to interpret facial expressions, body language or eye contact. Furthermore, not having to travel to a health care facility meant avoidance of sensory overload and mental exhaustion, sparing energy to focus on the treatment. To our knowledge, no previous studies have exhibited this pronounced advantage of IBT in relation to the needs of individuals with autism. This underlines the fit between the autistic difficulties and the IBT format.

However, by constantly striving to save energy, there is a risk that participants may also put less effort into the exercises and, thus may devaluate the importance of the treatment. Further, as noted by several participants, the treatment requires personal responsibility in planning and completing the exercises. However, the ability to plan and independently initiate activities is reduced among many with ASD. Accordingly, the limited support from a therapist provided in IBT may suit highly able individuals while being insufficient for others, which is in line with the findings of previous studies (45, 46, 57). In sum, providing sufficient support while simultaneously promoting independence is a difficult but important challenge for providers of IBT, and are important aspects to consider in the development of future programs.

Generally, the participants appreciated the text-based format. The possibility to contemplate a statement before delivering it was considered less stressful than providing immediate answers, as expected in a face-to face-situation. This finding confirms results from previous studies involving individuals with autism, suggesting a general need for increased time to formulate and express themselves (28, 52).

Furthermore, written communication did not affect the trust for, or contact with the therapist. However, the level of contact with the therapist varied greatly among the participants. Considering the diverse usage of the messaging service, it is a relevant question to ask whether the satisfaction with the treatment is affected by the level of therapist contact. A more in-depth analysis of the impact of therapist contact, as well as the content of messages between therapist and participant is warranted in future studies.

Most participants highlighted the importance of the therapists' knowledge about autism, and willingness to truly reach understanding of the patient. This presumably reflects a general desire for therapist engagement and competence within the field, as described by Nicolaidis (21) and is not exclusive to the online format. However, the preference of therapists with well-developed language and a direct communication style may reflect a wish among participants to avoid repeated experiences of previous misunderstandings in health care encounters. As the asynchronous online-communication offers little opportunities for immediate clarification of statements, an accessible and direct language is crucial to avoid misunderstandings.

The willingness to participate in the biweekly chat sessions varied between participants. The interpretation of our results is therefore based on the premise that some participants chose to not utilize the opportunity of peer interaction through the chats, which opens for the possibility of differences in evaluations between chat users and non-chat users. However, according to our results there are no pronounced differences in the evaluation of the program based on chat attendance. An analysis on potential associations between chat-participation and program satisfaction would be interesting for future quantitative analyses.

One reason for abstaining the chat sessions was fears or expected difficulties in socially interacting with others. This could be explained by low social self-esteem or negative experiences of social situations, both of which are common in this group. Further, ambiguities about the purpose of the chat, uncertainty as to what to expect, as well as beliefs about others' expectations of oneself may be reasons for abstaining the chat sessions. The unstructured design of the chat may also have been perceived as frightening for individuals with autism, who commonly need a structured setting with a clear objective to approach new situations (58).

Nevertheless, we are not aware of any other studies evaluating participant experiences of a group-based chat-session as part of an IBT for adults with ASD. The general perception among participants taking part in the chat was that they appreciated getting access to other perspectives. Chatting with others gave insights that they were not alone in their everyday struggles. The findings indicate that integrating a group-based component is feasible and offers an opportunity for social recognition, peer support and practicing communication skills from home. However, there is a possibility that providing a clearer rationale on the purpose and benefits of chatting would have increased participation. Consequently, one objective when integrating group-based components in IBT for people with ASD should be to establish a safe and predictable environment by offering a clear rationale and defining the purpose in advance. If accomplished, this would make the advantages of peer interaction available for as many patients as possible.

### Methodological Limitations and Data Quality

The quality of data is partly dependent on level of richness and depth in the participants' narratives. Due to mentalization deficits and phrasing difficulties, commonly observed in people with ASD, some of the narratives of this interview study lack in richness. Open-ended questions could be answered very briefly (“*it was good”*), or even with silence. This resulted in a tendency of the interviewer to narrow the questions to become more closed which poses a risk for influenced responses.

One limitation of the study is that only 10 participants had experience of the chat sessions, thus limiting the conclusions that can be drawn regarding peer interaction. Furthermore, the risk for selection bias must be addressed in relation to the interpretation of the data. It is an obvious issue that a purposive sampling method implies a certain bias—as those participants that had completed <8 modules were excluded from the study. Further, three participants were excluded due to current severe psychiatric comorbidity overshadowing the autistic traits. As shown in Table 3, 42.9 % of the participants in this study suffered from psychiatric comorbidity, compared with estimates of 54.8% in the population (4). In addition, several of the participants had children, a majority were referred to the ordinary labor market, and only one worked at a daily activity center (a municipally subsidized employment for people with disabilities).

The above reasons, along with the repeated comments on low complexity of exercises, may indicate that the participants in this study might be more highly functioning than average for people diagnosed with ASD. This may have resulted in a self-selection of high-functioning participants.

The interviewers had no relationship to the interviewees prior to the interview. However, all three interviewers were involved in the intervention—a fact known to the participants. Thus, both interviewee and interviewer may therefore have been biased toward more favorable evaluations.

## Conclusion

This qualitative interview study provides knowledge about how adults with ASD have experienced participation in an IBT. In general, the participants appreciated the flexibility and availability of the internet-based format, found that it facilitated their focus on treatment and regarded the written therapist interaction as safe and convenient. Further, our results suggests that integration of a group-based element is feasible, but a more thorough introduction and a more structured chat format may be needed to increase participation.

Overall, the internet-based format was well fitted to the needs of the participants in this study. However, the flexible framework requires a well-developed capacity to take responsibility over one's own treatment. Therefore, the internet-based format—as offered in the current intervention—may entail too little support for some individuals. In addition, several features of the treatment were perceived as not sufficiently adapted to individual needs or preferences.

The findings illustrate well-established knowledge—namely that there are substantial differences in needs, preferences, and functional abilities amongst individuals with ASD and normal intellectual abilities, despite having the same diagnosis. It also illustrates the importance of therapist support, suggesting that feedback is an important means to attain some level of individual tailoring.

Taking on a broader perspective, the possibility to acquire health care from distance have shown to be of great importance for the entire population considering the recent worldwide COVID 19-pandemic, and our study contributes to the knowledge on acceptability of such interventions among adults with ASD. Nevertheless, there is a need for future studies investigating ways of adapting IBT to better meet the diverse needs of adult individuals with ASD.

## Data Availability Statement

The datasets presented in this article are not readily available because ethical approval is mandatory. Requests to access the datasets should be directed to britta.westerberg@regionorebrolan.se.

## Ethics Statement

The studies involving human participants were reviewed and approved by the Regional Ethics Committee in Uppsala, Sweden (No. 2017/392). The patients/participants provided their written informed consent to participate in this study.

## Author Contributions

SBe conceived the original idea. BW, SBe, and FH designed the study. BW, SBe, and CG formed the interview guide and conducted the participant interviews. BW performed the analysis with support from SBe and UH. BW wrote the manuscript with input from all authors. CG reviewed and edited the manuscript. SBe, FH, and SBä supervised the project. All authors contributed to the interpretation of results and reviewed the final manuscript.

## Funding

This work was supported by the grant numbers of Region Örebro Län, Sweden (OLL-935396, OLL-879651, OLL-887401, OLL-833131, OLL-785501, OLL-785311, and OLL-736321) and Regional Research Council Mid Sweden (RFR-556731).

## Conflict of Interest

The authors declare that the research was conducted in the absence of any commercial or financial relationships that could be construed as a potential conflict of interest.

## Publisher's Note

All claims expressed in this article are solely those of the authors and do not necessarily represent those of their affiliated organizations, or those of the publisher, the editors and the reviewers. Any product that may be evaluated in this article, or claim that may be made by its manufacturer, is not guaranteed or endorsed by the publisher.

## Abbreviations

ADHD: attention deficit hyperactivity disorder
ASD: autism spectrum disorder
CBT: cognitive behavioral therapy
COREQ: consolidated criteria for reporting qualitative research
IBT: internet based treatment
ICBT: internet based cognitive behavioral therapy
IRL: in real life
MILAS: method for Internet based treatment for life quality for adults with autism spectrum disorder.

## References

[1] American Psychiatric Association. Diagnostic and Statistical Manual of Mental Disorders. 5th ed. Washington, DC: APA (2013).

[2] Murray C Kovshoff H Brown A Abbott P Hadwin JA. Exploring the anxiety and depression profile in individuals diagnosed with an autism spectrum disorder in adulthood. Res Autism Spectr Disord. (2019) 58:1–8. 10.1016/j.rasd.2018.11.002

[3] Hudson CC Hall L Harkness KL. Prevalence of depressive disorders in individuals with autism spectrum disorder: a meta-analysis. J Abnorm Child Psychol. (2019) 47:165–75. 10.1007/s10802-018-0402-1 29497980

[4] Lugo-Marín J Magán-Maganto M Rivero-Santana A Cuellar-Pompa L Alviani M Jenaro-Rio C. Prevalence of psychiatric disorders in adults with autism spectrum disorder: A systematic review and meta-analysis. Res Autism Spectr Disord. (2019) 59:22–33. 10.1016/j.rasd.2018.12.004

[5] Hollocks MJ Lerh JW Magiati I Meiser-Stedman R Brugha TS. Anxiety and depression in adults with autism spectrum disorder: a systematic review and meta-analysis. Psychol Med. (2019) 49:559–72. 10.1017/S0033291718002283 30178724

[6] Lever AG Geurts HM. Psychiatric co-occurring symptoms and disorders in young, middle-aged, and older adults with autism spectrum disorder. J Autism Dev Disord. (2016) 46:1916–30. 10.1007/s10803-016-2722-8 26861713 PMC4860203

[7] Gillberg IC Helles A Billstedt E Gillberg C. Boys with Asperger syndrome grow up: psychiatric and neurodevelopmental disorders 20 years after initial diagnosis. J Autism Dev Disord. (2016) 46:74–82. 10.1007/s10803-015-2544-0 26210519

[8] Hossain MM Khan N Sultana A Ma P McKyer ELJ Ahmed HU. Prevalence of comorbid psychiatric disorders among people with autism spectrum disorder: An umbrella review of systematic reviews and meta-analyses. Psychiatry Res. (2020) 287:112922. 10.1016/j.psychres.2020.112922 32203749

[9] Mason D McConachie H Garland D Petrou A Rodgers J Parr JR. Predictors of quality of life for autistic adults. Autism Res. (2018) 11:1138–47. 10.1002/aur.1965 29734506 PMC6220831

[10] Billstedt E Gillberg IC Gillberg C. Aspects of quality of life in adults diagnosed with autism in childhood: a population-based study. Autism. (2011) 15:7–20. 10.1177/1362361309346066 20923888

[11] Hirvikoski T Mittendorfer-Rutz E Boman M Larsson H Lichtenstein P Bolte S. Premature mortality in autism spectrum disorder. Br J Psychiatry. (2016) 208:232–8. 10.1192/bjp.bp.114.160192 26541693

[12] Lundström S Reichenberg A Anckarsäter H Lichtenstein P Gillberg C. Autism phenotype versus registered diagnosis in Swedish children: prevalence trends over 10 years in general population samples. BMJ. (2015) 350:h1961. 10.1136/bmj.h1961 25922345 PMC4413835

[13] Elsabbagh M Divan G Koh YJ Kim YS Kauchali S Marcín C. Global prevalence of autism and other pervasive developmental disorders. Autism Res. (2012) 5:160–79. 10.1002/aur.239 22495912 PMC3763210

[14] Calleja S Islam FMA Kingsley J McDonald R. Healthcare access for autistic adults: A systematic review. Medicine. (2020) 99:e20899. 10.1097/MD.0000000000020899 32702830 PMC7373620

[15] Tint A Weiss JA A. qualitative study of the service experiences of women with autism spectrum disorder. Autism. (2018) 22:928–37. 10.1177/1362361317702561 28914071

[16] Walsh C Lydon S O'Dowd E O'Connor P. Barriers to healthcare for persons with autism: a systematic review of the literature and development of a taxonomy. Dev Neurorehabil. (2020) 23:413–30. 10.1080/17518423.2020.1716868 36112897

[17] Weiss JA Lunsky Y. Group cognitive behaviour therapy for adults with Asperger syndrome and anxiety or mood disorder: a case series. Clin Psychol Psychother. (2010) 17:438–46. 10.1002/cpp.694 20827741

[18] Kerns CM Roux AM Connell JE Shattuck PT. Adapting cognitive behavioral techniques to address anxiety and depression in cognitively able emerging adults on the autism spectrum. Cogn Behav Pract. (2016) 23:329–40. 10.1016/j.cbpra.2016.06.002

[19] Hesselmark E Plenty S Bejerot S. Group cognitive behavioural therapy and group recreational activity for adults with autism spectrum disorders: a preliminary randomized controlled trial. Autism. (2014) 18:672–83. 10.1177/1362361313493681 24089423 PMC4230566

[20] Atkinson-Jones K Hewitt O. Do group interventions help people with autism spectrum disorder to develop better relationships with others? A critical review of the literature. Br J Learn Disabil. (2019) 47:77–90. 10.1111/bld.12258

[21] Nicolaidis C Raymaker DM Ashkenazy E McDonald KE Dern S Baggs AE. “Respect the way I need to communicate with you”: healthcare experiences of adults on the autism spectrum. Autism. (2015) 19:824–31. 10.1177/1362361315576221 25882392 PMC4841263

[22] Cassidy S Bradley L Shaw R Baron-Cohen S. Risk markers for suicidality in autistic adults. Mol Autism. (2018) 9:42. 10.1186/s13229-018-0226-4 30083306 PMC6069847

[23] White SW Simmons GL Gotham KO Conner CM Smith IC Beck KB. Psychosocial treatments targeting anxiety and depression in adolescents and adults on the autism spectrum: review of the latest research and recommended future directions. Curr Psychiatry Rep. (2018) 20:82. 10.1007/s11920-018-0949-0 30155584 PMC6421847

[24] Griffith GM Totsika V Nash S Hastings RP. 'I just don't fit anywhere': support experiences and future support needs of individuals with Asperger syndrome in middle adulthood. Autism. (2012) 16:532–46. 10.1177/1362361311405223 21610188

[25] Grynszpan O Weiss PL Perez-Diaz F Gal E. Innovative technology-based interventions for autism spectrum disorders: a meta-analysis. Autism. (2014) 18:346–61. 10.1177/1362361313476767 24092843

[26] Valencia K Rusu C Quinones D Jamet E. The impact of technology on people with autism spectrum disorder: a systematic literature review. Sensors. (2019) 19:4485. 10.3390/s19204485 31623200 PMC6832622

[27] van der Aa C Pollmann MMH Plaat A van der Gaag RJ. Computer-mediated communication in adults with high-functioning autism spectrum disorders and controls. Res Autism Spectr Disord. (2016) 23:15–27. 10.1016/j.rasd.2015.11.007

[28] Gillespie-Lynch K Kapp SK Shane-Simpson C Smith DS Hutman T. Intersections between the autism spectrum and the internet: perceived benefits and preferred functions of computer-mediated communication. Intellect Dev Disabil. (2014) 52:456–69. 10.1352/1934-9556-52.6.456 25409132

[29] Wright JH Owen JJ Richards D Eells TD Richardson T Brown GK. Computer-assisted cognitive-behavior therapy for depression: a systematic review and meta-analysis. J Clin Psychiatry. (2019) 80:18r12188. 10.4088/JCP.18r12188 30900849

[30] Stech EP Lim J Upton EL Newby JM. Internet-delivered cognitive behavioral therapy for panic disorder with or without agoraphobia: a systematic review and meta-analysis. Cogn Behav Ther. (2020) 49:270–93. 10.1080/16506073.2019.1628808 31303121

[31] Eilert N Enrique A Wogan R Mooney O Timulak L Richards D. The effectiveness of Internet-delivered treatment for generalized anxiety disorder: An updated systematic review and meta-analysis. Depress Anxiety. (2021) 38:196–219. 10.1002/da.23115 33225589 PMC7894171

[32] Kampmann IL Emmelkamp PM Morina N. Meta-analysis of technology-assisted interventions for social anxiety disorder. J Anxiety Disord. (2016) 42:71–84. 10.1016/j.janxdis.2016.06.007 27376634

[33] Pettersson R Soderstrom S Edlund-Soderstrom K Nilsson KW. Internet-based cognitive behavioral therapy for adults with ADHD in outpatient psychiatric care. J Atten Disord. (2017) 21:508–21. 10.1177/1087054714539998 24970720

[34] Westermann S Rüegg N Lüdtke T Moritz S Berger T. Internet-based self-help for psychosis: findings from a randomized controlled trial. J Consult Clin Psychol. (2020) 88:937–50. 10.1037/ccp0000602 32790453

[35] Conaughton RJ Donovan CL March S. Efficacy of an internet-based CBT program for children with comorbid high functioning autism spectrum disorder and anxiety: a randomised controlled trial. J Affect Disord. (2017) 218:260–8. 10.1016/j.jad.2017.04.032 28477505

[36] Khan K Hall CL Davies EB Hollis C Glazebrook C. The effectiveness of web-based interventions delivered to children and young people with neurodevelopmental disorders: systematic review and meta-analysis. J Med Internet Res. (2019) 21:e13478–e. 10.2196/13478 31682573 PMC6858614

[37] Carlbring P Andersson G Cuijpers P Riper H Hedman-Lagerlof E. Internet-based vs. face-to-face cognitive behavior therapy for psychiatric and somatic disorders: an updated systematic review and meta-analysis. Cogn Behav Ther. (2018) 47:1–18. 10.1080/16506073.2017.1401115 29215315

[38] Johansson R Andersson G. Internet-based psychological treatments for depression. Expert Rev Neurother. (2012) 12:861–9; quiz 870. 10.1586/ern.12.63 22853793

[39] Baumeister H Reichler L Munzinger M Lin J. The impact of guidance on Internet-based mental health interventions—a systematic review. Internet Interv. (2014) 1:205–15. 10.1016/j.invent.2014.08.003

[40] Fernández-Álvarez J Díaz-García A González-Robles A Baños R García-Palacios A Botella C. Dropping out of a transdiagnostic online intervention: a qualitative analysis of client's experiences. Internet Interv. (2017) 10:29–38. 10.1016/j.invent.2017.09.001 30135750 PMC6084825

[41] Arnold C Williams A Thomas N. Engaging with a web-based psychosocial intervention for psychosis: qualitative study of user experiences. JMIR Ment Health. (2020) 7:e16730. 10.2196/16730 32558659 PMC7334758

[42] Richards D Enrique A Palacios J Duffy D. Internet-delivered cognitive behaviour therapy. Cogn Behav Therapy Clin Appl. (2018) 187: 456–61. 10.5772/intechopen.71412

[43] Kraepelien M Forsell E Karin E Johansson R Lindefors N Kaldo V. Comparing individually tailored to disorder-specific internet-based cognitive-behavioural therapy: benchmarking study. BJPsych Open. (2018) 4:282–4. 10.1192/bjo.2018.41 30083380 PMC6066990

[44] Berger T Boettcher J Caspar F. Internet-based guided self-help for several anxiety disorders: a randomized controlled trial comparing a tailored with a standardized disorder-specific approach. Psychotherapy. (2013) 51:207–19. 10.1037/a0032527 24041199

[45] Jardine J Earley C Richards D Timulak L Palacios JE Duffy D. The experience of guided online therapy: a longitudinal, qualitative analysis of client feedback in a naturalistic RCT. In: Proceedings of the 2020 CHI Conference on Human Factors in Computing Systems (2020).

[46] Holst A Nejati S Bjorkelund C Eriksson MC Hange D Kivi M. Patients' experiences of a computerised self-help program for treating depression—a qualitative study of Internet mediated cognitive behavioural therapy in primary care. Scand J Prim Health Care. (2017) 35:46–53. 10.1080/02813432.2017.1288813 28277055 PMC5361419

[47] Lundgren J Johansson P Jaarsma T Andersson G Karner Kohler A. Patient experiences of web-based cognitive behavioral therapy for heart failure and depression: qualitative study. J Med Internet Res. (2018) 20:e10302. 10.2196/10302 30185405 PMC6231888

[48] Hughes S Sibelli A Everitt HA Moss-Morris R Chalder T Harvey JM. Patients' experiences of telephone-based and web-based cognitive behavioral therapy for irritable bowel syndrome: longitudinal qualitative study. J Med Internet Res. (2020) 22:e18691. 10.2196/18691 33216002 PMC7718092

[49] Rozental A Forsström D Tangen JA Carlbring P. Experiences of undergoing Internet-based cognitive behavior therapy for procrastination: a qualitative study. Internet Interv. (2015) 2:314–22. 10.1016/j.invent.2015.05.001

[50] Weisel KK Zarski A-C Berger T Krieger T Moser CT Schaub MP. User experience and effects of an individually tailored transdiagnostic internet-based and mobile-supported intervention for anxiety disorders: mixed-methods study. J Med Internet Res. (2020) 22:e16450–e16450. 10.2196/16450 32936085 PMC7527916

[51] Backman A Mellblom A Norman-Claesson E Keith-Bodros G Frostvittra M Bölte S. Internet-delivered psychoeducation for older adolescents and young adults with autism spectrum disorder (SCOPE): an open feasibility study. Res Autism Spectr Disord. (2018) 54:51–64. 10.1016/j.rasd.2018.07.001

[52] Sehlin H Hedman Ahlstrom B Andersson G Wentz E. Experiences of an internet-based support and coaching model for adolescents and young adults with ADHD and autism spectrum disorder—a qualitative study. BMC Psychiatry. (2018) 18:15. 10.1186/s12888-018-1599-9 29347983 PMC5774035

[53] Hsieh H-F Shannon S. Three approaches to qualitative content analysis. Qual Health Res. (2005) 15:1277–88. 10.1177/1049732305276687 16204405

[54] Bejerot S Björnstjerna E. ALMA—KBT för vuxna med autismspektrumsyndrom, manual och arbetsbok [ALMA-CBT for Adults with Autism Spectrum Disorder, Manual and Workbook]. (2019). Göttingen: Hogrefe Publishing.

[55] Eriksson JM Andersen LM Bejerot S. RAADS-14 Screen: validity of a screening tool for autism spectrum disorder in an adult psychiatric population. Mol Autism. (2013) 4:49. 10.1186/2040-2392-4-49 24321513 PMC3907126

[56] Tong A Sainsbury P. Craig, J. Consolidated criteria for reporting qualitative research (COREQ): a 32-item checklist for interviews and focus groups. Int J Qual Health Care. (2007) 19:349–57. 10.1093/intqhc/mzm042 17872937

[57] Walsh A Richards D. Experiences and engagement with the design features and strategies of an internet-delivered treatment programme for generalised anxiety disorder: a service-based evaluation. Br J Guid Counc. (2016) 45:16–31. 10.1080/03069885.2016.1153039

[58] National Institute for Health and Care Excellence. Clinical Guidelines (2016) Autism Spectrum Disorder in Adults: Diagnosis and Management. London: National Institute for Health and Care Excellence (UK) (2016).

